# Incidental Discovery of Persistent Müllerian Duct Syndrome in a Male With Bilateral Cryptorchidism and a Testicular Germ Cell Tumor: A Rare Case Report

**DOI:** 10.7759/cureus.79131

**Published:** 2025-02-17

**Authors:** Abdul Rauf Khalid, Sabtain Ali, Ghazanfar Ali, Muhammad Noor Ul Ul Huda, Faizan Shahzad, Abdulqadir J Nashwan

**Affiliations:** 1 Department of Surgery, Bahria International Hospital Orchard, Lahore, PAK; 2 Department of Surgery, Mayo Hospital, Lahore, PAK; 3 Department of Surgery, Holy Family Hospital, Rawalpindi, PAK; 4 Department of Medicine, Rawalpindi Medical University, Rawalpindi, PAK; 5 Department of Nursing and Midwifery Research, Hamad Medical Corporation, Doha, QAT

**Keywords:** cryptorchidism, histopathological evaluation, mixed germ cell tumor, persistent müllerian duct syndrome (pmds), testicular germ cell tumor (gct)

## Abstract

Cryptorchidism, or undescended testes, is a common congenital condition that significantly increases the risk of testicular malignancy, particularly germ cell tumors (GCTs), with a higher risk in the left testis. Persistent Müllerian Duct Syndrome (PMDS) is a rare disorder in males, where Müllerian structures such as a uterus or fallopian tubes are present despite the individual having a Y chromosome and male external genitalia. The concurrent occurrence of cryptorchidism, testicular tumors, and PMDS is extremely rare, making this case noteworthy. We report the case of a 24-year-old male with a history of bilateral cryptorchidism, who presented with an abdominal mass and intermittent pain. Imaging studies revealed a complex mass suggestive of a testicular tumor. During surgery, a small structure resembling a uterus was discovered and excised alongside both undescended testes. Histopathological evaluation confirmed a mixed GCT consisting of yolk sac tumor, seminoma, and teratoma arising from the undescended left testis. Additionally, the incidental finding of a uterine-like structure was consistent with PMDS. Postoperatively, the patient recovered without complications, with tumor markers normalizing within one month. Follow-up imaging and physical exams showed no recurrence at six months. This case highlights the rare association of PMDS with cryptorchidism and testicular tumors, emphasizing the importance of a multidisciplinary approach to diagnosis, treatment, and genetic counseling, particularly regarding fertility and associated conditions.

## Introduction

Cryptorchidism, or undescended testes, remains one of the most common congenital anomalies, affecting approximately 3-4% of full-term male infants. The condition significantly increases the risk of testicular malignancy, particularly germ cell tumors (GCTs), with a higher risk associated with the left testis [1-2]. Infertility is a frequent consequence of cryptorchidism, and testicular cancer is a major concern, particularly in untreated cases [2]. Persistent Müllerian Duct Syndrome (PMDS) is a rare congenital disorder of sexual differentiation in males, where individuals possess Müllerian structures such as a uterus or fallopian tubes despite having a Y chromosome and male external genitalia. This condition complicates the diagnosis and management of testicular tumors, making cases of testicular tumors occurring in conjunction with PMDS exceedingly rare and clinically significant [3]. Testicular tumors with both PMDS and cryptorchidism are exceedingly rare, making this case both an exceptional and clinically significant presentation. This report discusses a 24-year-old male with a history of bilateral cryptorchidism since birth, who presented with an abdominal mass that was subsequently diagnosed as a mixed GCT with an incidental finding of a small uterus, suggestive of PMDS.

## Case presentation

A 24-year-old male presented to our surgical outpatient department with complaints of a palpable mass in the left lower abdomen, associated with intermittent pain and a dragging sensation that had been present for the past few months. The patient denied any history of trauma or fever. He reported a growing feeling of fullness in the lower abdomen, which had progressively increased in size. Despite these symptoms, he had not experienced any changes in his urinary or gastrointestinal functions. The patient had bilateral cryptorchidism since birth but had not sought treatment for this condition, as it had not caused significant symptoms until recently. The patient had no history of significant illnesses, surgeries, or hospitalizations except for the known bilateral cryptorchidism. He had normal secondary sexual characteristics and underwent normal pubertal development without complications, indicating that both testes were functional despite being undescended.

Additionally, he had fathered two children, indicating preserved fertility. There was no significant family history of testicular cancer, cryptorchidism, or any other genetic conditions. On examination, the patient appeared well-nourished and was in no acute distress. Abdominal examination revealed a firm, non-tender mass of approximately 10 × 10 cm in the left lower quadrant, which was mobile. The rest of the systemic examination was unremarkable. There was no palpable testis in the scrotum, consistent with bilateral cryptorchidism. The genital and inguinal examinations revealed no additional abnormalities. Laboratory investigations (Table 1) showed an elevated serum beta-human chorionic gonadotropin (β-HCG) level of 1,470 IU/L (normal range: 0-5 IU/L), while alpha-fetoprotein (AFP) was within the normal range at 4 ng/mL (normal range: 0-10 ng/mL). LDH (lactate dehydrogenase) was also normal at 160 U/L (normal range: 140-280 U/L). Renal function tests (RFT) and complete blood count (CBC) were also within normal ranges.

**Table 1:** Laboratory test results for the patient with testicular germ cell tumor.

Test	Result	Normal Range
Serum Beta-Human Chorionic Gonadotropin (HCG)	1470 IU/L	0-5 IU/L
Alpha-fetoprotein (AFP)	4 ng/mL	0-10 ng/mL
Lactate Dehydrogenase (LDH)	160 U/L	140-280 U/L
Complete Blood Count (CBC)	Within normal limits	-
Renal Function Tests (RFT)	Within normal limits	-

**Figure 1:**
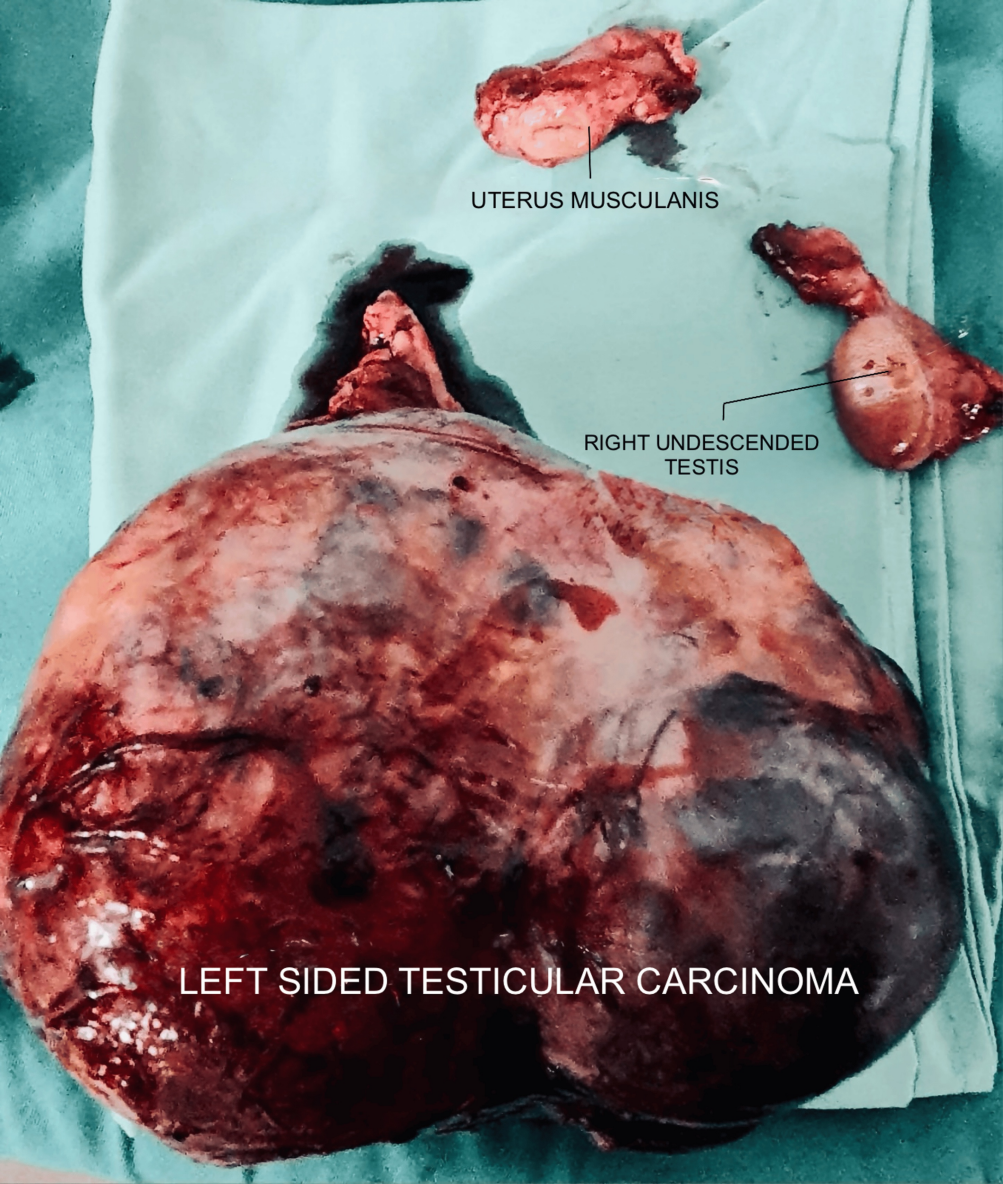
Intraoperative image showing the excised left testicular mass, an incidental uterus-like structure, and the right undescended testis, immediately post-excision before pathology evaluation.

A provisional diagnosis of a testicular tumor arising from an undescended left testis was made based on clinical findings and laboratory results. The elevated β-HCG level suggested a germ cell tumor. Diagnostic laparoscopy revealed a large mass in the left lower abdomen, but no testis was visualized. The mass appeared benign, with no adhesions or signs of malignancy. The left-sided mass was excised via midline laparotomy. Intraoperatively, both undescended testes were removed as part of the surgical procedure. During surgery, a small structure resembling a uterus was discovered in the pelvis, positioned between the bladder and rectum, and was also excised. The intraoperative findings, including the excised left testicular mass, the incidental uterus-like structure, and the right undescended testis immediately post-excision, are illustrated in Figure 1.

**Figure 2:**
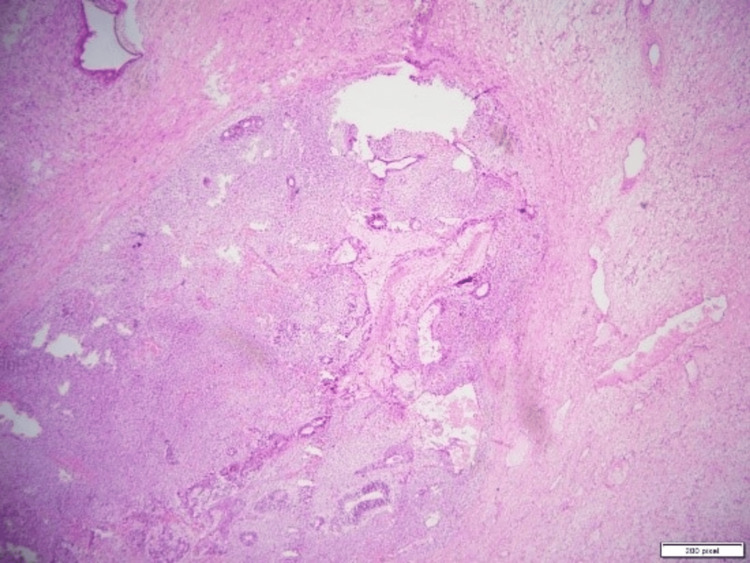
Histopathological specimen of the excised uterus-like structure, showing a small, rudimentary uterus with endometrial-like tissue.

**Figure 3:**
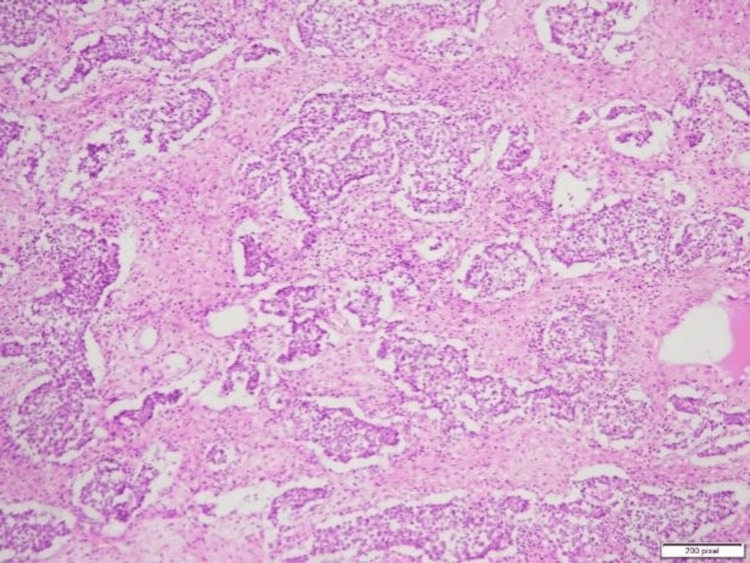
Histopathological specimen of the excised testicular mass, showing features consistent with seminoma, a type of germ cell tumor.

**Figure 4:**
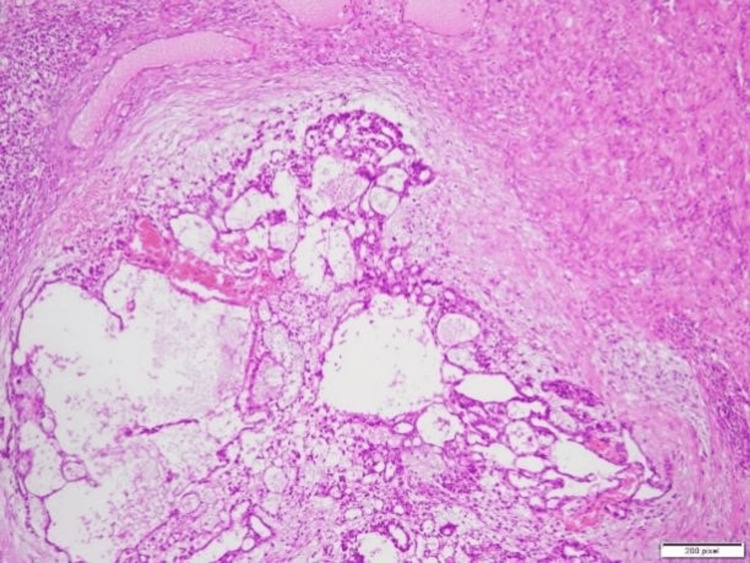
Histopathological specimen of the excised testicular mass, showing features consistent with a yolk sac tumor, a common component of mixed germ cell tumors.

**Figure 5:**
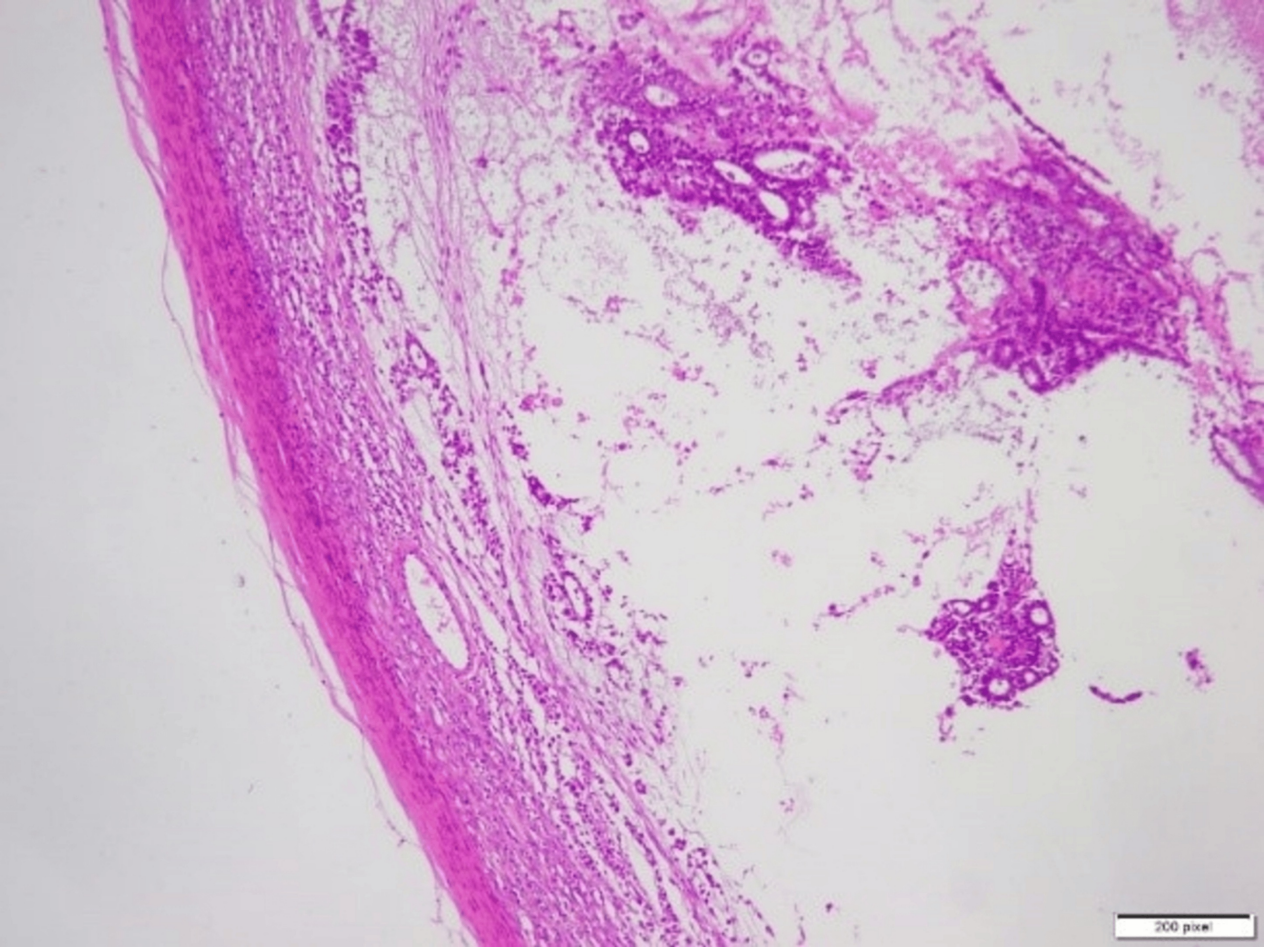
Histopathological specimen of the excised testicular mass, showing features consistent with a teratomatous component, a part of the mixed germ cell tumor.

The excised specimens, including the mass and the uterine-like structure, were sent for biopsy to determine their nature. The final diagnosis, based on biopsy results, confirmed a mixed germ cell tumor with components of a yolk sac tumor, seminoma, and teratoma arising from the undescended left testis. Additionally, the incidental finding of a small uterine-like structure in the pelvis, later identified as consistent with PMDS, was noted during pathological evaluation. The histopathological findings of the excised uterine-like structure (Figure 2) confirmed a rudimentary uterus. The testicular mass showed a seminomatous component (Figure 3), a yolk sac tumor with Schiller-Duval bodies (Figure 4), and a teratomatous component with differentiated tissues (Figure 5).

The patient had an uneventful recovery with no postoperative complications and was discharged with appropriate medications. For pain management, he was prescribed ibuprofen for five days. He was also given ceftriaxone, a broad-spectrum antibiotic, for one week to prevent infection. At his one-month follow-up visit, tumor markers had normalized (Table 2), and he continued to be closely monitored for recurrence. Additionally, genetic counseling was offered due to the incidental finding of PMDS.

**Table 2:** Postoperative tumor marker results.

Tumor Marker	Result postoperatively	Normal range
Serum Beta-Human Chorionic Gonadotropin (HCG)	5 IU/L	0-5 IU/L
Alpha-fetoprotein (AFP)	3 ng/mL	0-10 ng/mL
Lactate Dehydrogenase (LDH)	150 U/L	140-280 U/L

At six months, follow-up included normal physical examination findings and repeat imaging, which showed no recurrence of the mass. The patient continues to be monitored for any late recurrence or complications. Genetic counseling was provided due to the incidental finding of PMDS, and the patient was informed about the possible implications for fertility and other associated conditions. Regular follow-up visits are scheduled every three to six months for the next two years.

## Discussion

Cryptorchidism remains a significant risk factor for testicular cancer, particularly GCTs, which are the most common cancers in young men. This report presents a 24-year-old male with a history of bilateral cryptorchidism who developed a mixed GCT originating from an undescended left testis. His normal secondary sexual characteristics and fertility suggest functional compensation from the right testis. The incidental discovery of a small uterus during surgical exploration confirmed a diagnosis of PMDS, highlighting the importance of considering PMDS in cases of cryptorchidism with associated tumors. The risk of testicular cancer in individuals with cryptorchidism is significantly elevated, with studies estimating up to a 40-fold increase compared to those with normally descended testes [4]. Early surgical intervention, typically via orchiopexy before puberty, has been associated with a reduced risk of malignancy [5]. Moreover, long-term follow-up is necessary due to the potential for metachronous tumors and infertility issues. Although rare, testicular tumors in patients with PMDS require careful surgical and oncological management. Additionally, the diagnosis of PMDS should prompt clinicians to investigate other anomalies, including infertility and possible associations with syndromic conditions such as Wolfram syndrome. A multidisciplinary approach is recommended for management, including surgical excision of abnormal structures and genetic counseling to guide reproductive planning and further investigation of associated abnormalities [2].

This patient’s discovery of a small uterus during surgery adds complexity to the case and highlights the importance of considering PMDS when dealing with cryptorchidism and associated tumors. A key limitation of this case is the lack of a detailed neonatal history, which restricts insights into early-life associations with PMDS. Although rare, testicular tumors in patients with PMDS are possible, and they require careful surgical and oncological management. Furthermore, the diagnosis of PMDS should alert clinicians to other possible anomalies, including infertility and a higher likelihood of other associated conditions, such as Wolfram syndrome. To our knowledge, no previous case has been reported with the simultaneous presence of Persistent Müllerian Duct Syndrome, bilateral cryptorchidism, and a mixed GCT. This highlights the rarity of this condition and underscores the need for increased awareness among clinicians managing cryptorchidism and testicular tumors. Management requires a multidisciplinary approach, including surgical excision of abnormal structures and genetic counseling to guide family planning and further investigation of associated abnormalities.

## Conclusions

with bilateral cryptorchidism and a testicular germ cell tumor is an exceptionally rare occurrence. This case underscores the importance of considering PMDS in patients with cryptorchidism and testicular tumors, as incidental findings can influence surgical and management decisions. Early diagnosis through imaging and genetic counseling is crucial for optimizing patient outcomes. Further research is needed to explore the genetic basis of PMDS and its potential long-term implications, aiding in better clinical management and early detection strategies.
